# Benthic Oxygen Uptake in the Arctic Ocean Margins - A Case Study at the Deep-Sea Observatory HAUSGARTEN (Fram Strait)

**DOI:** 10.1371/journal.pone.0138339

**Published:** 2015-10-14

**Authors:** Cecile Cathalot, Christophe Rabouille, Eberhard Sauter, Ingo Schewe, Thomas Soltwedel

**Affiliations:** 1 Laboratoire des Sciences du Climat et de l’Environnement, UMR CEA/CNRS/UVSQ, Gif-sur-Yvette, France; 2 Alfred-Wegener-Institut I Helmholtz-Zentrum für Polar-und Meeresforschung, Bremerhaven, Germany; Auckland University of Technology, NEW ZEALAND

## Abstract

The past decades have seen remarkable changes in the Arctic, a hotspot for climate change. Nevertheless, impacts of such changes on the biogeochemical cycles and Arctic marine ecosystems are still largely unknown. During cruises to the deep-sea observatory HAUSGARTEN in July 2007 and 2008, we investigated the biogeochemical recycling of organic matter in Arctic margin sediments by performing shipboard measurements of oxygen profiles, bacterial activities and biogenic sediment compounds (pigment, protein, organic carbon, and phospholipid contents). Additional *in situ* oxygen profiles were performed at two sites. This study aims at characterizing benthic mineralization activity along local bathymetric and latitudinal transects. The spatial coverage of this study is unique since it focuses on the transition from shelf to Deep Ocean, and from close to the ice edge to more open waters. Biogeochemical recycling across the continental margin showed a classical bathymetric pattern with overall low fluxes except for the deepest station located in the Molloy Hole (5500 m), a seafloor depression acting as an organic matter depot center. A gradient in benthic mineralization rates arises along the latitudinal transect with clearly higher values at the southern stations (average diffusive oxygen uptake of 0.49 ± 0.18 mmol O_2_ m^-2^ d^-1^) compared to the northern sites (0.22 ± 0.09 mmol O_2_ m^-2^ d^-1^). The benthic mineralization activity at the HAUSGARTEN observatory thus increases southward and appears to reflect the amount of organic matter reaching the seafloor rather than its lability. Although organic matter content and potential bacterial activity clearly follow this gradient, sediment pigments and phospholipids exhibit no increase with latitude whereas satellite images of surface ocean chlorophyll *a* indicate local seasonal patterns of primary production. Our results suggest that predicted increases in primary production in the Arctic Ocean could induce a larger export of more refractory organic matter due to the longer production season and the extension of the ice-free zone.

## Introduction

The Arctic Ocean plays an important role in many biogeochemical atmospheric and marine cycles [1, 2]. Although seasonal sea-ice cover moderates the gas exchange between the atmosphere and the ocean, the Arctic Ocean contributes for 5–14% to the global CO_2_ budget [3]. Future Climate Change feedbacks affecting the Arctic Ocean are hence expected to severely impact not only local carbon budgets but also global marine carbon cycle [3, 4]. Sea-ice loss, ocean warming, changes in salinity [5, 6], and shifts in nutrient distributions will affect carbon fluxes in the Arctic Ocean [7], directly changing the structure and functioning of marine ecosystems [8–10]. For instance, significant shifts in the microbial food webs have been observed in 2007 in the Amundsen Gulf (Beaufort Sea) after the massive loss of multi-year sea ice [10]. Similarly, reductions in sea ice cover in marginal seas of the Canadian Arctic are driving shifts in marine species composition and carbon cycling, including changes in zooplankton composition and species range extensions of benthic fauna [11]. Such changes might lead to a weakening of the benthic—pelagic coupling in the Arctic Ocean with serious consequences to an already food-limited benthic food web [12]. In particular, sea-ice retreat in the Arctic Ocean can lead to a decrease in the labile detritus flux [13, 14]. Consequences to the benthic carbon cycle are poorly documented [13] and a better understanding of the interplay between sea-ice oscillations and export of particulate organic carbon (POC) and its mineralization processes at the Arctic seafloor is still needed.

As the ultimate receptacle of the organic carbon export flux from surface waters, the seafloor acts as a habitat for benthic fauna and bacteria. On short time scales the benthic degradation of organic matter in the sediment regenerates inorganic carbon and nutrients. [15]. Benthic carbon degradation processes consist in a complex sequence of oxidation pathways with a succession of electron acceptors including O_2_, NO_3_^−^, Mn and Fe-oxides, SO_4_^2-^, organic matter and CO_2_ [15, 16]. The relative importance of the different pathways depends on the quality of the settling material, the sediment type and the rain rate of particulate organic carbon (POC). In the open ocean and with increasing water depth, O_2_ generally becomes dominant [15, 17]. Part of the total benthic oxygen consumption is used for the re-oxidation of reduced products originating from anaerobic mineralization [18–21]. Hence, the benthic O_2_ uptake is a commonly used measure for the total benthic mineralization rate [22]. The diffusive part of the benthic O_2_ uptake rates can be inferred from the O_2_ concentration gradients measured across the sediment—water interface, which enable the calculation of the diffusive oxygen uptake (DOU) rates as a proxy for the benthic carbon degradation activity [23, 24]. O_2_ respiration by benthic fauna and transport of oxic water into their burrows also affect the oxygen distribution in the sediment and hence carbon mineralization processes. The total oxygen uptake (TOU), measured by incubating sediment, accounts for both microbial (inferred mostly through DOU) and faunal processes [22, 25].

The deep-sea observatory HAUSGARTEN offers a unique window on the biogeochemistry of the Arctic Ocean and is an ideal site to detect and track environmental changes in an ice-edge region. Located in the Fram Strait, it lies in the transition zone between the northern North Atlantic and the central Arctic Ocean. Characterized by the inflow of relatively warm and nutrient-rich Atlantic Water into the central Arctic Ocean, hydrographic features in the Fram Strait lead to permanent ice-covered areas in the west, permanent ice-free areas in the south-east, and seasonal varying ice conditions in the central and north-eastern parts that form the HAUSGARTEN area [26]. Bioproduction in the area is directly coupled to these sea-ice conditions. Indeed, the melting of ice in spring and summer leads to a stratified euphotic Marginal Ice Zone (MIZ), which is rich in nutrients and causes intense phytoplankton blooms (up to 30 mg C m^-2^ d^-1^) and regionally enhanced fluxes of particulate organic matter to the seafloor [27]. Over the past decades, the sea-ice extent has been shrinking causing a northward shift of the ice-edge and its associated primary production, with direct incidence to the export flux of particulate organic matter (POM) to the seafloor and the associated benthic carbon cycle.

**Fig 1:**
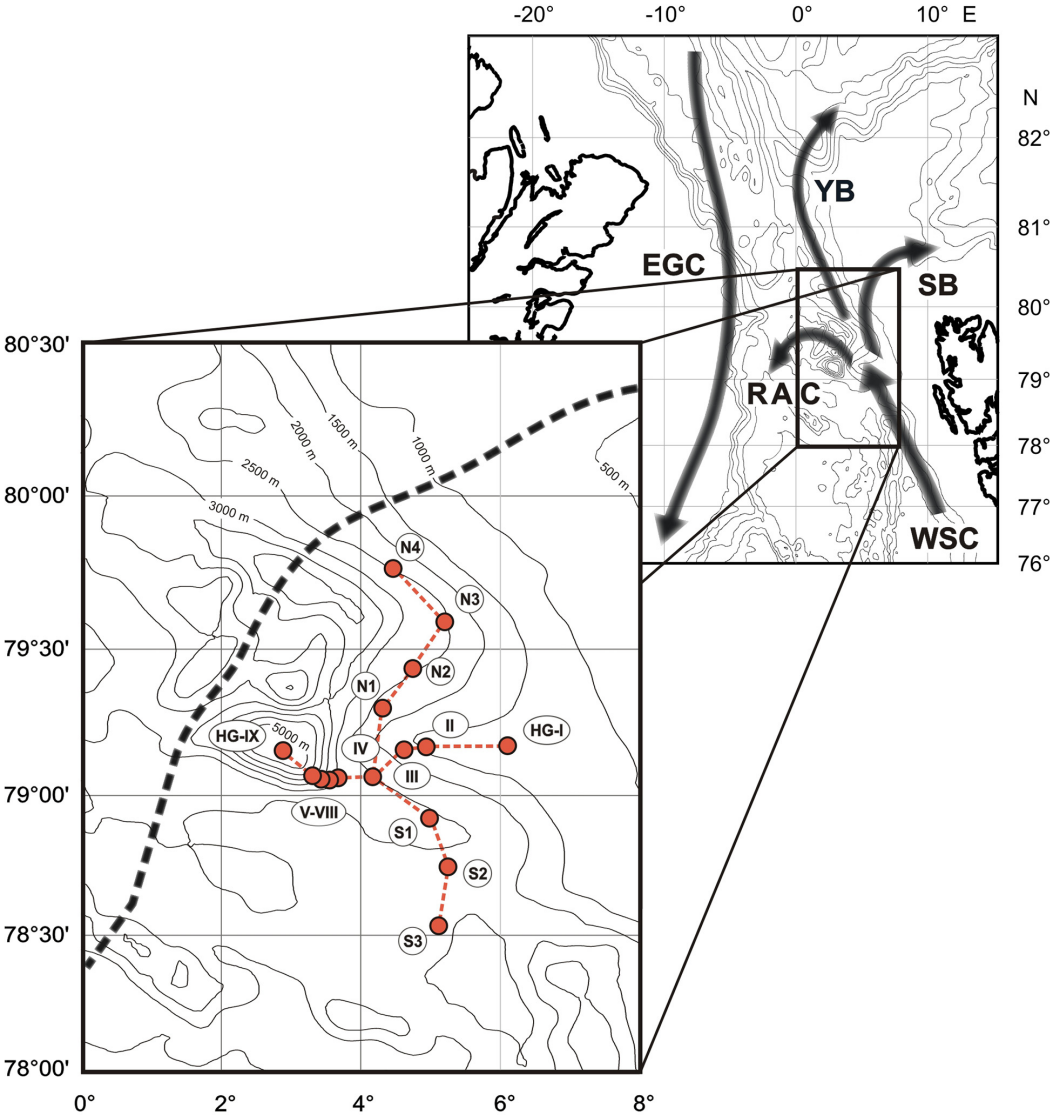
Map of the Fram Strait. Black arrows indicate the main circulation patterns. Insight focuses on the HAUSGARTEN area. Red symbols indicate the sampling stations and the dotted black line represent the average ice-edge limit during summer (i.e. minimum sea ice extent).

The present study aims at characterizing benthic mineralization along both the bathymetric transect and the latitudinal transect at the HAUSGARTEN observatory (Fig 1). The data set consists of benthic O_2_ uptake rates quantified from O_2_ concentration profiles obtained both in the laboratory and *in situ* during summer 2007 and 2008. Rather than focusing on the temporal variability inherent to the region, our study offers a spatial coverage of DOU rates in the Fram Strait at the interface between two regimes: i) from the continental shelf to the abyss, and ii) from the Atlantic to the Polar regime across the sea-ice front. Such DOU compilation is quite unique in the Arctic environments as existing datasets generally focus in either the shelf or the deep ocean [28]. Our results help constraining the benthic carbon cycle in the Arctic Ocean margin and we discuss the effect of the ice-edge zone on the benthic degradation activity.

## Material and Methods

### Site description

The deep-sea observatory HAUSGARTEN is located in the Fram Strait, the only deep water connection between the central Arctic Ocean and the Nordic Seas [26]. The observatory includes 16 sampling sites positioned along a bathymetric transect between 1200 and 5500 m water depth (stations KH, Kb0, HGI, HGII, HGIII, HGIV, HGVI, HGVII, HGVIII and HGIX) and along a latitudinal transect following the 2500 m isobath (stations N4, N3, N2, N1, S2 and S3) crossing each other at the central HAUSGARTEN site (Fig 1).

The bathymetry in the study area is characterized by the Vestnesa Ridge, a submarine projection from the Svalbard continental margin (approximately 1000–2000 m water depth), and the Molloy Hole, a deep depression with a maximum depth of 5669 m [29], adjoining the ridge to the west (stations HGVIII and HGIX). Steepest parts of the slope reach up to 40° inclination in water depths between 4000 and 5000 m. Surface sediments in the HAUSGARTEN area are dominated by silioclastic components that are supplied by sea ice, downslope transport from the Svalbard shelf and advection from distal source areas.

The hydrographic conditions in the HAUSGARTEN area are characterized by the inflow of relatively warm and nutrient-rich Atlantic Water (AW) into the central Arctic Ocean [30]. Approximately 20% of the northward flowing AW (West Spitsbergen Current, WSC) is re-circulated within the Return Atlantic Current (RAC) at 78–80°N (Fig 1). At about the same latitude, the remaining AW splits up into the Svalbard Branch (33% of WSC waters), following the perimeter of the Svalbard archipelago, and the Yermak Branch (45% of WSC waters), flowing along the western and northern flanks of the Yermak Plateau, a sub-sea peninsula to the northwest of Svalbard. The cooler less-saline Polar Water exits the central Arctic Ocean as the Eastern Greenland Current (EGC) in western parts of the Fram Strait, separated by a frontal system (East Greenland Polar Front) from the water masses at HAUSGARTEN observatory.

### Biogenic sediment compounds

Sediment samples taken by a multiple corer (MUC) with internal diameter of 8 cm during expeditions in 2007 and 2008 were analyzed for various biogenic sediment compounds indicating sediment porosity, organic matter content and lability, and bacterial activity. Subsamples were taken for biochemical analyses with 5- and 20-mL disposable syringes with cut-off tips. Generally, three pseudo-replicates from each MUC (randomly sampled from different MUC tubes) were analyzed to estimate small-scale variations within the area covered by the MUC.

Porosity was estimated by measuring the weight loss of wet sediment samples dried at 60°C, and assuming a sediment density of 2.5 g cm^-3^. The total organic matter content of the sediments was determined as ash-free dry weight after combustion (2 h, 500°C), and reported as the organic carbon content (%C Org, dw). The bioavailability of phytodetritus at the seafloor was assessed by measuring sediment-bound chlorophyll *a* (Chl *a*) and its degradation products, pheopigments (Pheo). Chloroplastic pigments were extracted in 90% acetone and measured with a TURNER fluorometer [31]. The bulk of pigments (chlorophyll *a* plus pheopigments) was termed chloroplastic pigment equivalents, CPE [32]. The ratio of Chl *a*: total chloroplastic pigment content (%Chl *a*) serves as an indicator for the relative “lability” of the phytodetrital matter. Phospholipids, indicating the quantity of cell wall material, were measured following Findlay et al. [33], as modified by Boetius and Lochte [34]. Particulate proteins (referred hereafter as proteins), which were operationally defined as γ-globulin equivalents [35], were measured to estimate the bulk of “living” and “dead biomass”, i.e. organisms and the proportion of detrital organic matter in the sediments. Bacterial enzymatic turnover rates were determined using the fluorogenic substrate fluorescein-di-acetate (FDA) as an indicator of the potential hydrolytic activity of bacteria [36].

For all parameters, average values for the uppermost 5 cm of the sediment were considered.

### Diffusive oxygen uptake (DOU) rates

Sediment cores for *ex situ* microprofiler measurements were sampled using the MUC at various HAUSGARTEN stations along the latitudinal and bathymetric (across the shelf down the slope) transect (Table 1). Immediately after recovery, an aliquot of the water overlying the sediment was sampled to determine oxygen concentration by Winkler titration [37]. Measurements of O_2_ microprofiles were performed in the cold room at *in situ* temperature (approx. 0°C). Up to three O_2_ microprofiles per core were completed with a resolution of 0.5 to 1 mm within 6 h after sampling. The O_2_ microsensors had tip outer diameters of 50–100 μm, a stirring sensitivity of <1%, a 90% response time <10 s, and less than 2% per hour current drift. Conservation of overlying water oxygenation was achieved by a soft bubbling system. In July 2007, microprofiles were performed at 12 stations in the HAUSGARTEN area: six along the bathymetric transect across the shelf down the slope (HGI, HGIV, HGVI, HGVII, HGVIII, HGIX) and another six along the latitudinal transect (N4, N3, N2, N1, S2, S3). In July 2008, microprofiles were also obtained along both transects, respectively at stations HGI, HGII, HGIII, HGIV, HGV, HGVI, and stations S1, S2, S3, N3. In addition, two more coastal stations, KH and Kb0, were sampled to extend the bathymetric transect for DOU measurements towards Svalbard (Table 1): porosity was assumed to be 0.85 at these two sites. A total of six stations were sampled both years.

**Table 1:** Sampling stations at the HAUSGARTEN site.

HAUSGARTEN station	Station number	Sampling date	Latitude	Longitude	Depth
***Bathymetric transect***					
I	PS70/163-1	12/07/2007	79° 08.07' N	05° 59.45' E	1304
	PS72/137-2	12/07/2008	79° 08.00' N	06° 05.51' E	1287
II	PS70/159-1	11/07/2007	79° 07.82' N	04° 53.65' E	1565
	PS72/136-2	11/07/2008	79° 07.78' N	04° 53.91' E	1562
III	PS70/151-1	11/07/2007	79° 06.15' N	04° 34.81' E	2037
	PS72/131-2	11/07/2008	79° 06.47' N	04° 36.30' E	1943
IV	PS70/147-1	10/07/2007	79° 03.92' N	04° 10.55' E	2477
	PS72/122-2	09/07/2008	79° 03.92' N	04° 11.01' E	2417
V	PS70/183-1	15/07/2007	79° 03.92' N	03° 41.86' E	2990
	PS72/141-3	12/07/2008	79° 03.21' N	03° 44.74' E	2861
VI	PS70/184-1	15/07/2007	79° 03.60' N	03° 34.81' E	3548
	PS72/142-2	12/07/2008	79° 02.78' N	03° 36.00' E	3442
VII	PS70/211-1	19/07/2007	79° 03.59' N	03° 28.50' E	4065
	PS72/160-1	17/07/2008	79° 03.50' N	03° 28.83' E	4070
VIII	PS70/212-1	19/07/2007	79° 03.79' N	03° 18.80' E	5140
IX	PS70/222-1	22/07/2007	79° 08.21' N	02° 50.77' E	5590
	PS72/159-2	17/07/2008	79° 08.56' N	02° 45.61' E	5503
***Supplementary stations extending the bathymetrical transect to the East***					
Kb0	PS72/106-2	06/07/2008	79° 01.70' N	11° 05.20' E	287
KH	PS72/118-3	08/07/2008	79° 01.79' N	06° 59.93' E	1312
***Latitudinal transect***					
S1	PS70/179-1	15/07/2007	78° 55.10' N	04° 59.90' E	2641
	PS72/125-2	10/07/2008	78° 55.00' N	05° 00.06' E	2636
S2	PS70/175-1	14/07/2007	78° 46.85' N	05° 19.98' E	2477
	PS72/126-2	10/07/2008	78° 46.81' N	05° 19.98' E	2465
S3	PS70/174-1	13/07/2007	78° 36.54' N	05° 03.82' E	2354
	PS72/129-3	10/07/2008	78° 36.47' N	05° 03.77' E	2342
N1	PS70/193-1	16/07/2007	79° 16.97' N	04° 19.70' E	2406
N2	PS70/194-1	16/07/2007	79° 24.57' N	04° 41.80' E	2552
	PS72/147-2	15/07/2008	79° 25.57' N	04° 44.65' E	2597
N3	PS70/197-1	17/07/2007	79° 36.32' N	05° 09.23' E	2804
	PS72/146-1	14/07/2008	79° 35.69' N	05° 12.47' E	2772
N4	PS70/200-1	17/07/2007	79° 44.19' N	04° 25.66' E	2644
	PS72/145-3	14/07/2008	79° 44.18' N	04° 29.40' E	2694

In addition, *in situ* 500-μm-resolution O_2_ profiles were obtained at station S3 and HGIV by a benthic microprofiler (Unisense®) equipped with 4–5 O_2_ microelectrodes. The autonomous profiling unit was handled by a work-class Remotely Operated Vehicle (ROV). Dissolved oxygen concentration was measured by oxygen microelectrodes (Unisense®) provided with a built-in reference and an internal guard cathode [38]. The O_2_ microsensors were similar to those used in the cold room. The electrode signals were recorded in the overlying water before and after each profile to assess the stability of the measurements. We used a linear calibration for the microelectrodes, between the bottom-water oxygen content estimated by Winkler titration [37] and the anoxic zone of the sediment when reached or the signal in an anoxic solution of sodium ascorbate.

We assigned the sediment—water interface location to a break in the oxygen concentration gradient. The observed change of slope is due to the decreased diffusion coefficient in the sediment compared to the diffusive boundary layer [38].

Oxygen profiles were used to calculate the diffusive oxygen uptake (DOU) rate of the sediment. The diffusive oxygen fluxes at the sediment—water interface (SWI) were assessed using Fick’s first law:

$$DOU=\;-\emptyset \;Ds\;{\left[\frac{\partial O_{2}}{\partial z}\right]}_{z=0},$$

where Ds is the molecular diffusion coefficient in sediments at *in situ* temperature, ${\left[\frac{\partial O_{2}}{\partial z}\right]}_{z=0}$ is the oxygen gradient at the SWI determined by linear regression over the 5 top mm of the sediment (selected as the minimum interval to cover the linear section of all gradients) and Ø is the porosity of the sediment.

Ø was derived from the water content data and Ds was calculated using Archie’s law as Ds = D_0_ Ø^2.5^, where D_0_ is the molecular diffusion coefficient [39].

### Statistical treatment

All statistical analyses for sediment compounds and water content were performed using the average values of the 0–5 cm top sediment layer.

Statistical differences between sampling years for the following sediment parameters were tested using the non-parametric Wilcoxon signed rank-sum test: porosity, organic carbon content (C org), Chl *a*, Pheo, protein and phospholipid contents, bacterial exo-enzymatic activity (FDA) and DOU rates. Hence, for each parameter and at each station, pair-wise comparisons between year 2007 and 2008 were used to assess statistical differences.

Along the latitudinal transect, the overall relationships between DOU and the main biogeochemical parameters (CPE, %Chl *a*, organic carbon, phospholipid and protein contents, and FDA) of the surface sediments (0–5 cm top layer) were assessed using a Spearman correlation matrix and a principal component analysis (PCA). CPE and %Chl *a* were chosen as variables over Chl *a* and Pheo contents, in order to have both qualitative (%Chl *a*) and quantitative (CPE) parameters of labile organic matter in the PCA.

All statistical tests and procedures were run on R Statistical Software (version 2.14.0; R Foundation for Statistical Computing, Vienna, Austria).

### Ethics Statement

During these sampling procedures, no permits were required as we only collected sediment samples that were collected in international waters. The HAUSGARTEN observatory is part of the European Network of Excellence ESONET (European Seas Observatory Network) and the Infrastructure Projekt EMSO (European Multidisciplinary Seafloor Observatory), and is also a member of the LTER (Long-Term Ecological Research) Network.

## Results

### Sediment parameters

Environmental parameters along the bathymetric transect generally decreased with increasing station depth (Table 2). Except for phospholipids in 2007 and particulate proteins as well as organic carbon content in 2008, highest values were always found at the shallowest HAUSGARTEN station HGI (~1290 m water depth). For most of the sediment parameters, lowest values were analysed for station HGVII (~4068 m). Almost all parameters showed increased values at HGVIII (~5140 m) and HGIX (~5540 m), situated on the continental rise and in the central Molloy Deep, respectively. Higher values at the deepest stations were in the range of those found for mid-water depths (~2000–3000 m) along the bathymetric transect (Table 2).

**Table 2:** Sediment characteristics and biogenic sediment compounds in surface sediment at the HAUSGARTEN site (Ø, porosity; Chl *a*, Chlorophyll *a* content; Pheo, Pheopigments content; Protein, Protein content readily soluble per sediment volume; C Org, Organic Carbon content; Lipid, Phospholipids content; FDA, esterase activity per sediment volume; DOU, Diffusive Oxygen Uptake rates). Avg: average value over the upper 5 cm sediment. Std: standard deviation.

Station	Year	Ø	Chl *a*		Pheo		Proteins		C org		Lipids		FDA		DOU	
			*μg cm* ^*-3*^		*μg cm* ^*-3*^		*mg cm* ^*-3*^		*%*		*μg C cm* ^*-3*^		*nmol mL* ^*-1*^ *h* ^*-1*^		*mmol O*_*2*_ *m* ^*-2*^ *d* ^*-1*^	
		*avg*	*avg*	*std*	*avg*	*std*	*avg*	*std*	*avg*	*std*	*avg*	*std*	*avg*	*std*	*avg*	*std*
***Bathymetric transect***																
**HGI**	2007	0.84	3.7	1.57	23.8	4.53	2.1	0.11	7.6	0.00	11.8	3.48	5.0	2.28	0.87	0.06
	2008	0.83	2.5	1.41	27.9	8.58	1.0	0.05	9.5	0.07	35.6	8.70	3.8	0.74	0.87	0.00
**HGII**	2007	0.81	2.7	0.43	21.0	11.57	0.4	0.05	-	-	11.4	2.21	3.0	0.65	-	-
	2008	0.81	1.8	0.29	17.8	2.81	0.6	0.07	5.6	0.11	16.4	3.10	2.9	0.30	0.95	0.02
**HGIII**	2007	0.77	3.3	1.06	23.0	3.45	0.4	0.03	6.9	0.45	17.7	3.18	3.0	0.42	-	-
	2008	0.75	2.2	1.48	23.3	10.99	1.3	0.05	5.0	0.87	30.7	4.68	2.6	0.29	0.45	-
**HGIV**	2007	0.72	2.0	0.66	11.9	2.95	0.5	0.18	4.1	0.73	8.6	2.01	3.1	0.65	0.28	0.07
	2008	0.72	0.7	0.22	12.1	3.42	0.5	0.06	4.3	1.42	18.6	4.63	1.8	0.40	0.38	-
**HGV**	2007	0.69	1.8	1.32	13.8	3.47	0.3	0.04	4.7	0.75	15.7	4.14	1.0	0.21	-	-
	2008	0.72	1.1	0.19	11.2	2.85	0.9	0.19	4.8	0.60	34.9	5.95	1.2	0.15	0.55	-
**HGVI**	2007	0.65	1.5	0.27	11.3	0.98	0.5	0.11	3.8	0.51	8.6	4.08	0.8	0.08	0.34	0.03
	2008	0.70	0.8	0.21	8.6	0.72	0.5	0.07	6.5	0.59	15.8	9.53	1.2	0.11	0.73	0.03
**HGVII**	2007	0.57	0.9	0.49	6.8	4.82	0.5	0.05	2.6	0.32	2.8	0.91	0.4	0.09	0.06	0.00
	2008	0.54	0.5	0.11	6.3	1.25	0.3	0.06	2.5	0.40	21.2	9.73	0.4	0.02	-	-
**HGVIII**	2007	0.63	1.7	1.25	11.8	8.16	2.0	1.43	2.3	0.04	3.9	1.64	0.2	0.11	0.69	0.13
	2008	-	-	-	-	-	-		-	-	-		-		-	-
**HGIX**	2007	0.81	-	-	-	-	0.4	0.01	5.7	0.89	8.7	3.03	0.3	0.03	0.41	0.06
	2008	0.79	0.9	0.21	12.1	2.33	1.4	0.15	6.9	0.69	16.5	3.83	0.3	0.05	-	-
***Supplementary stations extending the bathymetrical transect to the East***																
Kh	2008	0.85*	-	-	-	-	-	-	-	-	-	-	-	-	0.63	0.04
Kb0	2008	0.85*	-	-	-	-	-	-	-	-	-	-	-	-	2.49	0.11
***Latitudinal transect***																
**N4**	2007	0.67	1.7	0.31	9.4	2.52	1.1	0.16	2.5	0.40	6.3	1.49	1.3	0.19	0.14	0.03
	2008	0.67	0.7	0.16	7.3	1.55	0.9	0.04	3.9	0.05	9.0	3.33	1.5	0.16	-	-
**N3**	2007	0.77	1.7	1.22	11.3	2.99	0.3	0.03	5.0	1.50	16.6	6.59	1.5	0.35	0.22	0.02
	2008	0.75	0.9	0.20	10.5	2.42	0.6	0.07	3.6	0.32	22.8	4.59	2.3	0.46	0.20	-
**N2**	2007	0.79	2.2	0.24	16.1	2.19	0.4	0.01	6.0	1.24	16.4	5.12	1.4	0.64	0.37	0.05
	2008	0.78	1.4	0.74	12.5	1.83	0.4	0.02	4.1	0.49	32.4	6.06	1.9	0.37	-	-
**N1**	2007	0.70	1.9	0.93	15.0	5.95	0.3	0.04	4.8	0.22	13.5	5.42	1.8	0.46	0.18	0.01
	2008	-	-	-	-	-	-	-	-	-	-	-	-	-	-	-
**HGIV**	2007	0.72	2.0	0.66	11.9	2.95	0.5	0.18	4.1	0.73	8.6	2.01	3.1	0.65	0.28	0.07
	2008	0.72	0.7	0.22	12.1	3.42	0.5	0.06	6.5	0.59	18.6	4.63	1.8	0.40	0.38	-
**S1**	2007	0.80	2.2	0.27	16.9	3.29	1.4	0.02	6.2	1.09	13.1	2.51	2.0	0.51	-	-
	2008	0.78	1.2	0.48	14.0	3.41	0.5	0.02	5.4	0.95	4.9	7.39	2.1	0.28	0.42	-
**S2**	2007	0.74	1.5	1.06	9.4	3.86	0.4	0.10	5.7	1.39	11.2	3.49	1.8	0.38	0.82	0.08
	2008	0.75	0.9	0.18	9.8	2.91	0.4	0.08	5.2	0.82	21.4	5.46	2.4	0.23	0.57	0.05
**S3**	2007	0.75	2.0	0.56	11.5	3.03	0.2	0.02	5.6	1.05	6.1	3.03	1.9	0.59	0.38	0.01
	2008	0.76	0.8	0.32	11.1	1.01	1.1	0.02	5.8	1.13	16.6	6.74	2.5	0.25	0.56	0.02

* assumed porosity values (for DOU calculations purposes).

Sediment porosities ranged between 0.84 at HGI in 2007 and 0.54 at HGVII in 2008. Maximum Chl *a* concentrations of 3.7 μg cm^-3^ were found at HGI in 2007, whereas lowest values of 0.5 μg cm^-3^ were determined for station HGVII in 2008. Pheo concentrations ranged between 27.9 μg cm^-3^ at HGI and 6.3 μg cm^-3^ at HGVII, both in 2008. Highest protein contents of 2.1 mg cm^-3^ were found at HGI in 2007, whereas lowest concentrations of 0.3 mg cm^-3^ were analysed at HGVII in 2008. Maximum organic carbon contents (C Org) of 9.5% were also found at HGI, whereas lowermost values of 2.3 were determined at HGVIII, both in 2008. Phospholipid values ranged between 35.6 μg C cm^-3^ at HGI in 2008 and 2.8 μg C cm^-3^ at HGVII in 2007. Highest potential exo-enzymatic activity (FDA) of 5.0 nmol mL^-1^ h^-1^ was found at HGI, whereas the lowest activity of 0.2 nmol mL^-1^ h^-1^ was determined for HGVIII, both in 2007.

Along the latitudinal transect, there was no near clear trend in sediment porosity and Pheo concentrations (7.3–9.4 μg cm^-3^), which showed lowermost values in sediments at the northernmost site N4 (Table 2). In contrast, sediments at N4 exhibited overall higher protein contents (0.9–1.1 mg cm^-3^). Except for bacterial exo-enzymatic activity (FDA) and C Org contents, there was no clear trend with distance from the ice-edge situated in northern parts of the HAUSGARTEN observatory. Mean FDA and C Org values at the northern stations, however, were on average slightly lower compared to values on the southern stations HGIV and S1-S3 (Table 2).

### Diffusive oxygen uptake (DOU)

**Fig 2:**
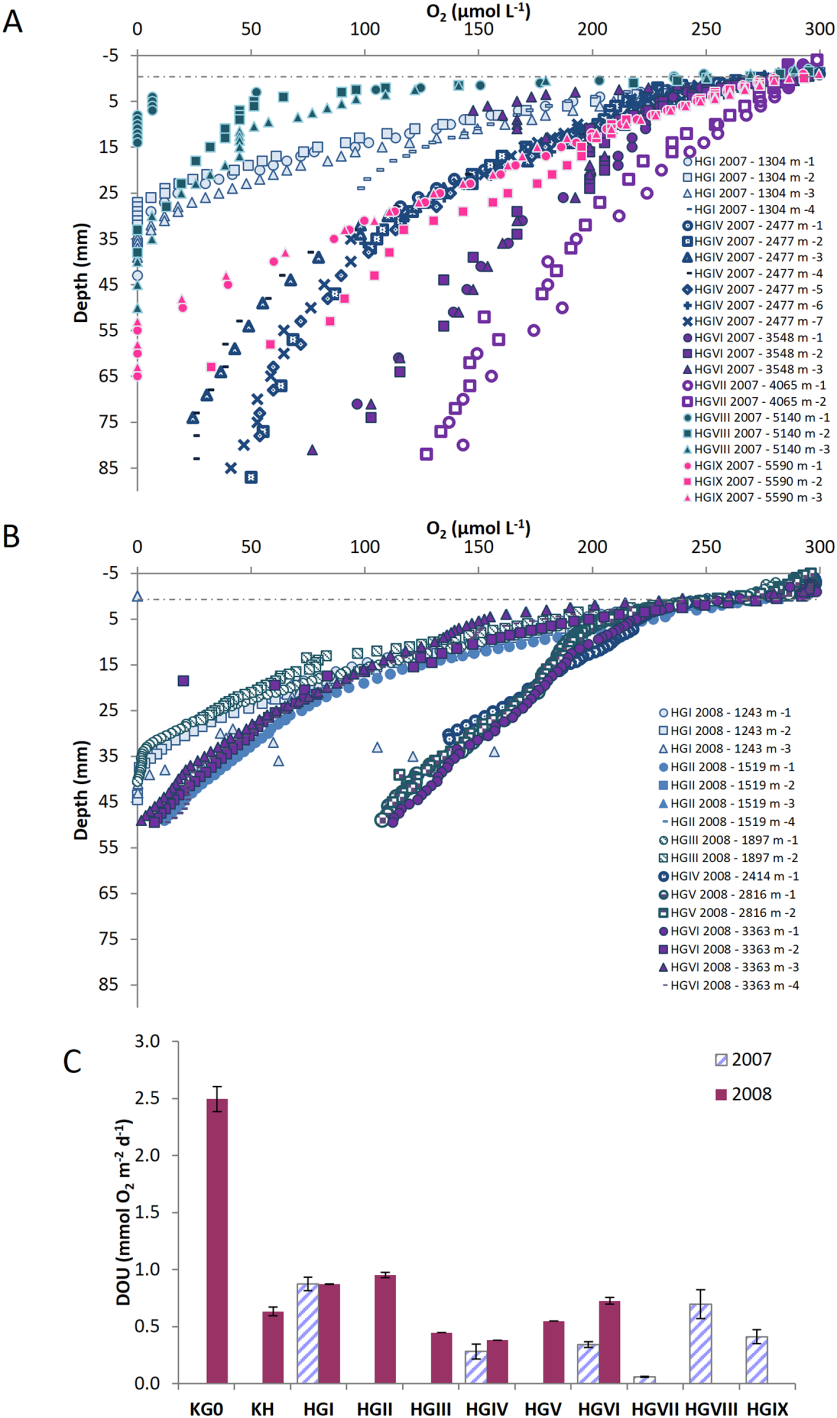
Sediment oxygen microprofiles and diffusive sediment oxygen uptake along the bathymetric transect. (A) Oxygen microprofiles obtained along the bathymetric transect in 2007. (B) Oxygen microprofiles obtained along the bathymetric transect in 2008. Numbers in the legend indicate the profile number. (C) Diffusive sediment oxygen uptake along the bathymetric transect for both years.

**Fig 3:**
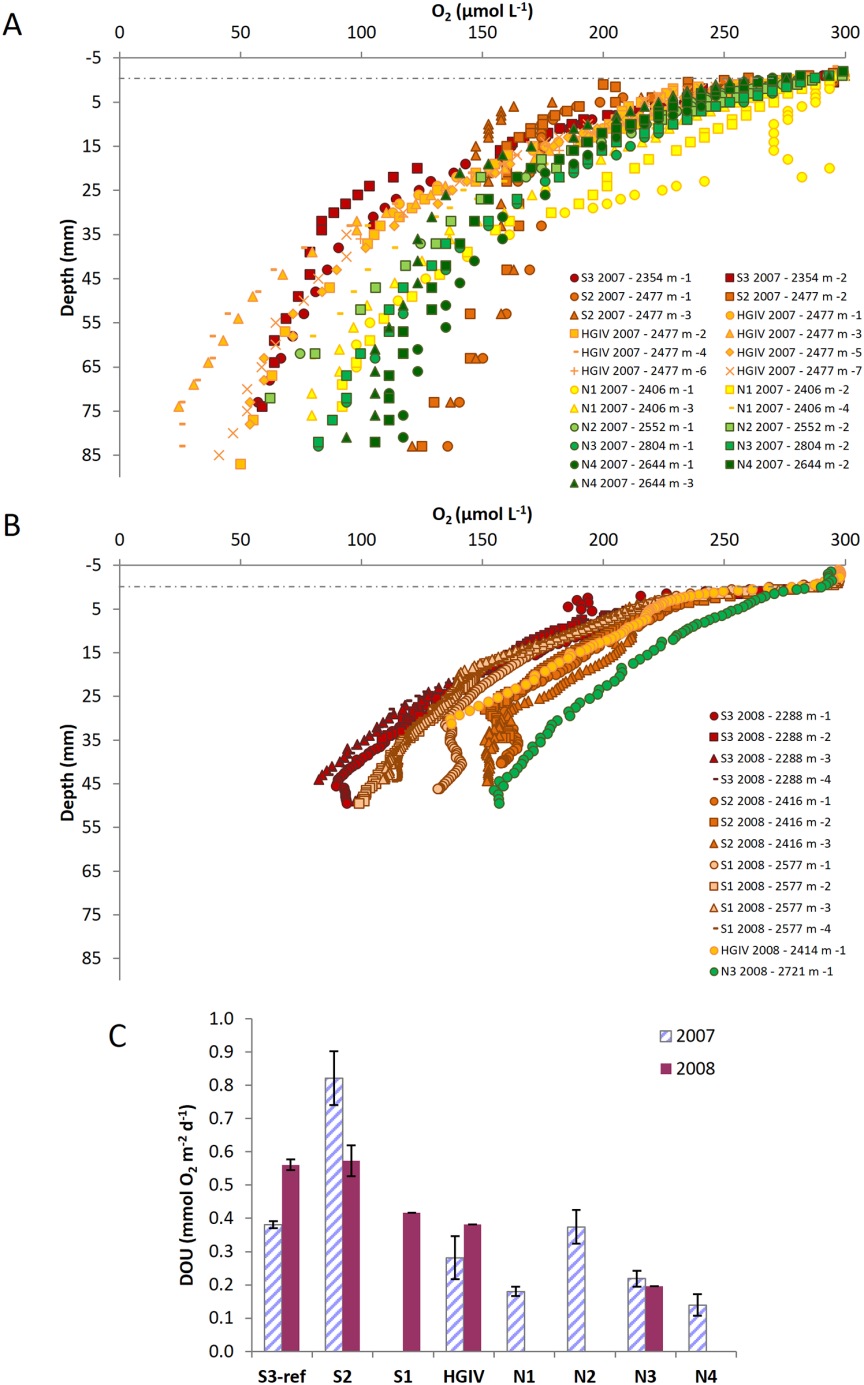
Sediment oxygen microprofiles and diffusive sediment oxygen uptake along the North-South transect. (**A**) Oxygen microprofiles obtained along the North-South transect in 2007. (**B**) Oxygen microprofiles obtained along the North-South transect in 2008. Numbers in the legend indicate the profile number. (**C**) Diffusive sediment oxygen uptake along the North-South transect for both years.

All oxygen profiles showed decreasing O_2_ concentrations below the sediment—water interface, the steepness of the O_2_ gradient depending on the station (Figs 2 and 3, Table 2).

The sediment oxygen penetration depth (OPD) is visible on a restricted set of profiles from the shallowest stations (Kb0, KH, HGI) and Molloy Hole stations (HGVIII, HGIX), where it ranges from 7 mm at the shallowest station (Kb0) to 55 mm at HGIX. Apart from these stations, oxygen in the porewater was present down to 60–85 mm, our maximum electrode penetration.

Along the bathymetric transect, DOU decrease from the eastern-most and shallowest station (Kb0) to station HGVII, with values of 2.49 and 0.06 mmol O_2_ m^−2^ d^−1^, respectively. DOU increase at stations HGVIII and HGIX located on the lower slope and in the Molloy Hole (Fig 2). Along the latitudinal transect, DOU ranged between 0.14 and 0.82 mmol O_2_ m^−2^ d^−1^, with higher values at the southern stations (Table 2, Fig 3).

At the central station (HGIV), DOU derived from both *in situ* and *ex situ* microprofiling gave almost similar values (0.20 ± 0.03 mmol O_2_ m^−2^ d^−1^ for *in situ* measurements and 0.28 ± 0.07 mmol O_2_ m^−2^ d^−1^ for *ex situ* profiles). At S3, another *in situ* vs *ex situ* comparison was performed which provided similar results (values of 0.29 ± 0.04 vs 0.38 ± 0.01 mmol O_2_ m^−2^ d^−1^, respectively).

### Statistical results

Except for Chl *a* and phospholipid contents that exhibit respectively higher and lower values in 2007, no significant differences in DOU and sediment parameters were found between 2007 and 2008 (Table 3). Moreover, along the latitudinal transect only Chl *a* displayed significant differences between both years. Both data sets were therefore pooled together to study spatial patterns.

**Table 3:** *p*-values of Wilcoxon rank-sum test for statistical differences of variables between years 2007 and 2008 (Chl *a*., chlorophyll *a* content; Pheo, pheopigments content; Protein, protein content readily soluble per sediment volume; C Org, organic carbon content; Lipid, phospholipids content; FDA, esterase activity per sediment volume; DOU, diffusive oxygen uptake). Values in bold and italic indicate *p*-values < 0.05.

	*p value*
**porosity**	0.5594
**C Org**	0.9460
**Chl *a***	***0*.*0002***
**Pheo**	0.0681
**Protein**	0.7536
**Lipids**	***0*.*0017***
**Fda**	0.5301
**DOU**	0.5896

Along the bathymetric transect DOU showed significantly positive correlations with pigments contents (Table 4).

**Table 4:** Pearson correlations coefficients calculated between water depth, biogenic sediment compounds (values for the 0–5 cm layer), and diffusive oxygen uptakes rates for stations along the bathymetric transect at the HAUSGARTEN site (C Org, organic carbon content; Chl *a*, chlorophyll *a* content; Pheo, pheopigments content; Protein, protein content readily soluble per sediment volume; Lipid, phospholipids content; FDA, esterase activity per sediment volume; Depth, water depth; DOU, diffusive oxygen uptake).

	Porosity	Chl *a*	Pheo	Protein	C Org	Lipid	FDA	Depth	DOU
**Porosity**	1.00								
**Chl *a***	**0.60** *	1.00							
**Pheo**	**0.87** ***	**0.83** ***	1.00						
**Protein**	0.32	0.53	0.46	1.00					
**C Org**	**0.92** †	0.42	**0.75** **	0.27	1.00				
**Lipid**	**0.60** *	0.57	0.82	0.08	0.52	1.00			
**FDA**	**0.72** **	0.70	**0.77** **	-0.10	**0.62** *	**0.73** **	1.00		
**Depth**	**-0.63** *	**-0.77** **	**-0.73** **	0.07	-0.50	**-0.68** **	**-0.98** †	1.00	
**DOU**	0.47	**0.73** **	0.55	0.44	0.17	0.28	0.42	-0.48	1.00

* p < 0.1,

** p < 0.05

*** p < 0.01

^†^ p < 0.001

**Fig 4:**
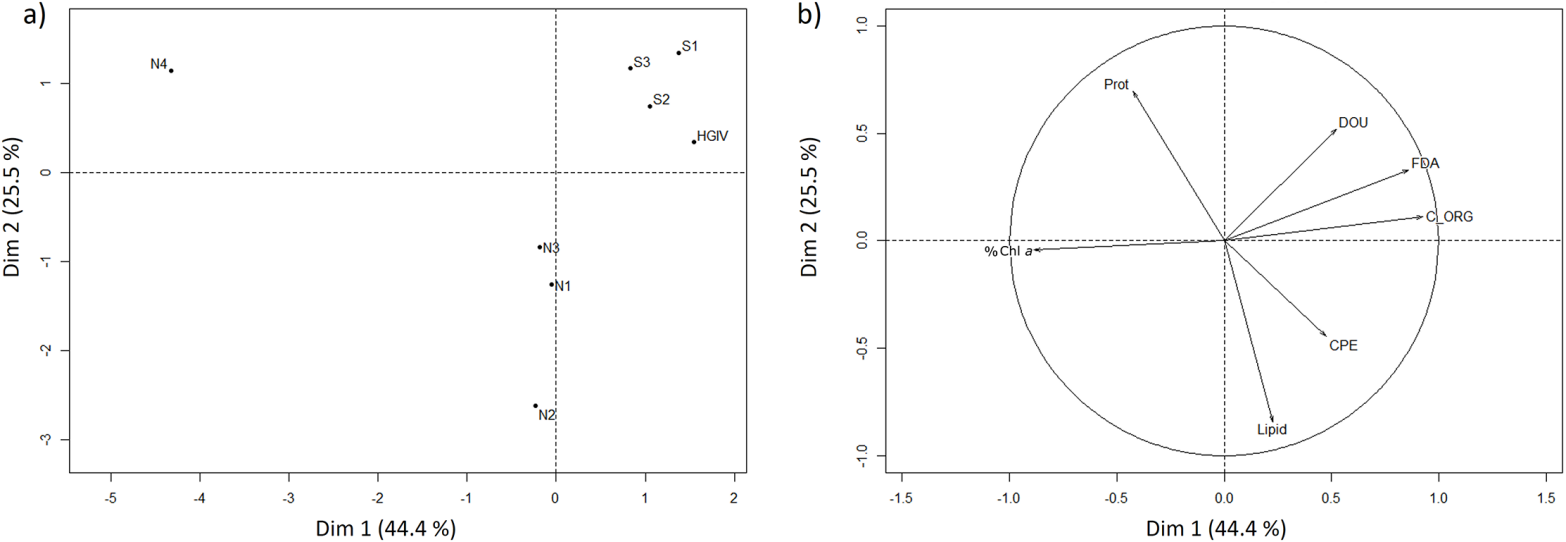
Projections of the HAUSGARTEN stations along the NS transect (A) and variables (B) on the first plane of a PCA based on sedimentary organics. Qualitative descriptors of organic matter and diffusive oxygen uptake measured at each sampled stations (C_ORG, organic carbon content; FDA, esterase activity; CPE, chloroplastic pigment equivalents;% Chl *a*, ratio of Chl *a* from the total chloroplastic pigment content; Lipid, phospholipid content; Prot, protein content; DOU, diffusive oxygen uptake)

Along the latitudinal transect DOU showed a strong positive correlation with organic carbon content in the sediment (Table 5). C Org, FDA, and DOU correlated negatively with latitude. The projections of variables on the two first planes of the PCA are shown in Fig 4. The first two components accounted for 44.4% and 25.5% of total variance, respectively. Component 1 was associated with quantitative sediment characteristics, which correlated positively with DOU, AFDW, FDA, and negatively with %Chl *a* contents. Component 2 was more associated with qualitative biogenic compounds, namely protein, phospholipid and Pheo contents, these two lasts being negatively correlated.

**Table 5:** Pearson correlations coefficients calculated between depth, surface sediments properties, sedimentary organics, diffusive oxygen uptakes rates along the latitudinal transect at the HAUSGARTEN site (C Org, organic carbon content; Chl *a*, chlorophyll *a* content; Pheo, pheopigments content; Protein, protein content readily soluble per sediment volume; Lipid, phospholipids content; FDA, esterase activity per sediment volume; DOU, diffusive oxygen uptake).

	Porosity	Chl *a*	Pheo	Protein	C Org	Lipid	FDA	Depth	DOU
**Porosity**	1.00								
**Chl *a***	0.36	1.00							
**Pheo**	0.47	**0.90** ***	1.00						
**Protein**	-0.02	-0.40	-0.24	1.00					
**C Org**	0.57	0.23	0.45	0.12	1.00				
**Lipid**	0.39	0.11	0.02	**-0.72** **	-0.12	1.00			
**FDA**	0.14	-0.11	0.10	0.05	**0.69** *	0.05	1.00		
**Depth**	0.14	-0.42	-0.29	0.32	-0.45	0.07	-0.52	1.00	
**DOU**	0.20	-0.34	-0.14	0.08	**0.67** *	-0.10	**0.71** **	-0.24	1

* p < 0.1

** p < 0.05

*** p < 0.01.

Northern and southern stations were clearly apart, indicating different regimes between northern and southern sites. Southern stations were associated with higher organic matter content (C Org), respiration rates (DOU) and bacterial exo-enzymatic activity (FDA). Northern stations were more characterized by higher Pheo and phospholipid contents.

## Discussion

### 2007 and 2008: comparable years for biogeochemical parameters

Over the summers 2007 and 2008, the spatial pattern of benthic carbon mineralization in the HAUSGARTEN area was rather stable. Indeed, for stations that have been sampled both years, no significant differences in DOU were observed (p > 0.5), and we conclude that a similar pattern of DOU in the sediments occurred for both years.

**Fig 5:**
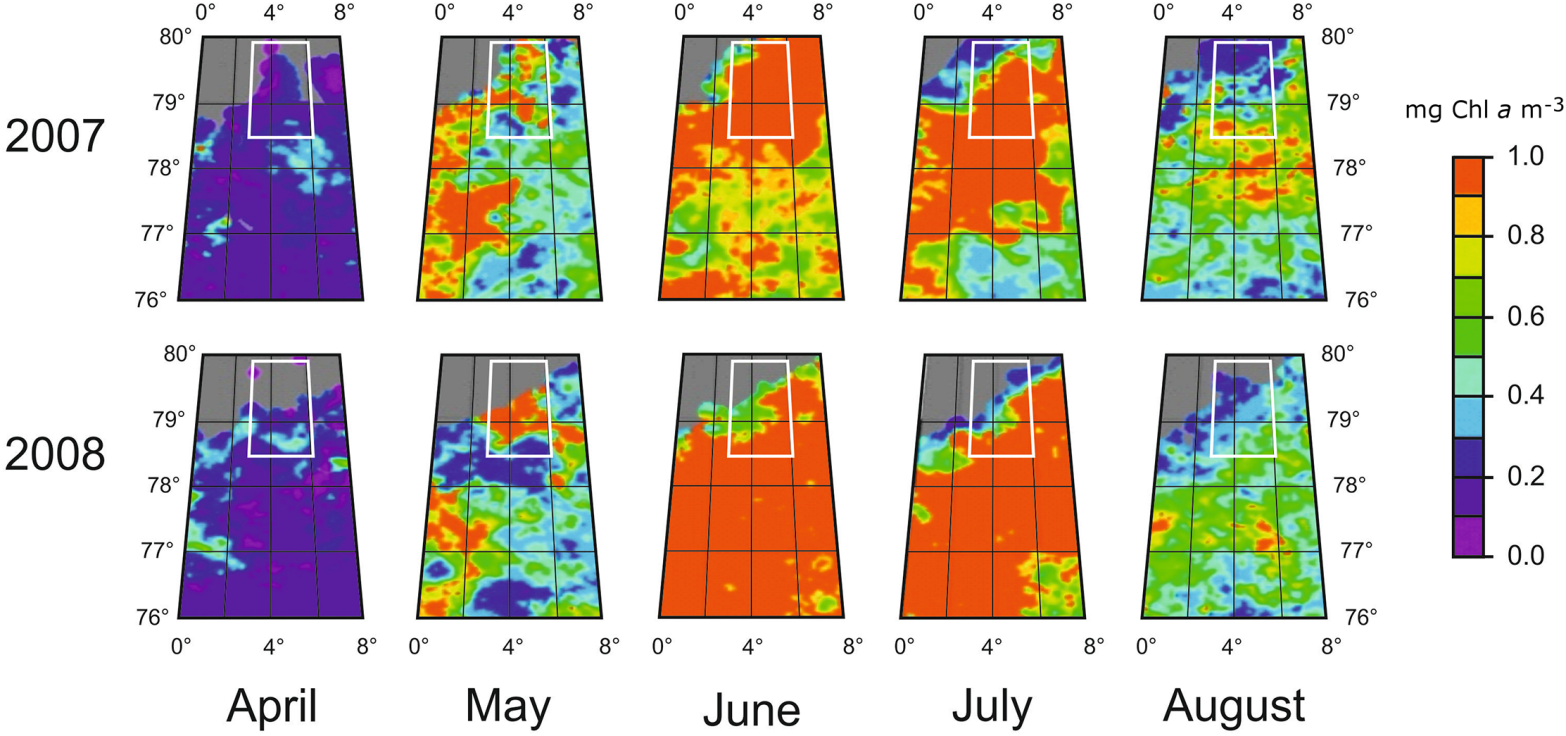
Surface water Chl *a* in 2007 and 2008 in the wider HAUSGARTEN region. The maps figuring Global Colour Chlorophyll a were obtained from a merged MODIS-MERIS-SeaWiFS product. The aerial coverage is 76°–80°N and 0°–8°E and the spatial resolution is 4.6 km. The temporal resolution is 8 days to monthly. The grey area represents ice-covered regions.

Benthic communities in Arctic sediments are limited by carbon availability rather than by low temperatures and/or hydrostatic pressure [40]: hence, such similarity in the benthic mineralization pattern between the two years suggests comparable inputs of organic matter to the seafloor in 2007 and 2008. As a matter of fact, satellite images (MODIS data) of the surface chlorophyll *a* development in the wider HAUSGARTEN area in 2007 and 2008 showed similar situations for both years (Fig 5), indicating that surface primary production in summer was equivalent in quantity and similar in spatial pattern for 2007 and 2008. Also, hydrographic data from moored instruments, showed comparable flow and circulation patterns of Atlantic waters in the Fram Strait for both years [41]; ICES, 2012). Moreover, sediment trap data in the HAUSGARTEN area showed similar POC flux at ~250 m depth between 2007 and 2008 with values around 20 mg C m^-2^ d^-1^ [42]. In addition, except for the Chl *a* and phospholipid contents, which were otherwise in the typical range for Arctic sediment values at these depths [43], all biogeochemical sediment descriptors analyzed in this study showed no significant differences between both years, suggesting similar particulate organic matter (POM) fluxes reaching the seafloor, in quantitative terms but different in quality. Higher Chl *a* contents and “lability” of the phytodetritial matter (%Chl *a*) observed in 2007, and higher phospholipids contents measured in 2008 point towards a higher quality of POM reaching the seafloor in 2007 [44], along with a delayed reaction of the microbial community. Likely, the phytoplankton community in 2008 might have suffered from some limitations (nutrients or light) and/or the late fall bloom might have dominated the POM export flux [45, 46], resulting in a shift in the phytoplankton production or in the community itself. In 2007, a shorter time lag between the settling of phytodetritus and the time of our sediment sampling could explain further part of the higher Chl *a* and phospholipid contents observed that year in comparison with 2008. Besides suggesting different qualitative origins of the surface primary production and since benthic mineralization rates are similar during both years, the differences in Chl *a* contents (and therefore %Chl *a* ratios) and phospholipid contents of the sediments observed between 2007 and 2008 also points towards a less labile organic matter reaching the seafloor between the two years, both Chl *a* and phospholipid contents being proxies for organic matter lability [44]. Although primary production in Polar Seas exhibits large variations in space and time [47], it is likely that the export of primary production from the surface waters at the HAUSGARTEN area was similar in 2007 and 2008 but with different reactivity between both years.

It is commonly assumed that sediment receive organic matter can be separated in three types: 1) “labile” organic matter that is rapidly hydrolizable and completely mineralized regardless of redox conditions, 2) a less degradable organic material embedded in a mineral matrix (multi or mono-layer coating) that degrades slowly in presence of oxygen but not under anoxic conditions and 3) refractory organic matter [48]. Sediments in the Arctic Ocean are characterized by a deep oxygen penetration (Figs 2 and 3) and dominated by the degradation of intermediate-reactivity organic matter [49]. Hence, the flux of the most labile part of organic matter reaching the seafloor is often negligible. In our study, such low flux of labile organic matter is indicated by the low Chl *a* content of surface sediments (Table 2), which is a common proxy for phytodetritus inputs [50, 51]. DOU of HAUSGARTEN sediment remains constant over both years while the Chl *a* content varied: rather than being controlled by the substrate type as it has been shown previously [52], DOU in the HAUSGARTEN site seems to be driven by the slow oxic degradation of the less degradable fraction of organic matter. Likely, benthic mineralization activity in the HAUSGARTEN area is dominated by a less degradable fraction of organic matter, which is less subjected to yearly variation and has time to degrade completely at its intrinsic rate until only refractory organic material remains [48, 49].

### Benthic mineralization activity along the bathymetric HAUSGARTEN transect

**Fig 6:**
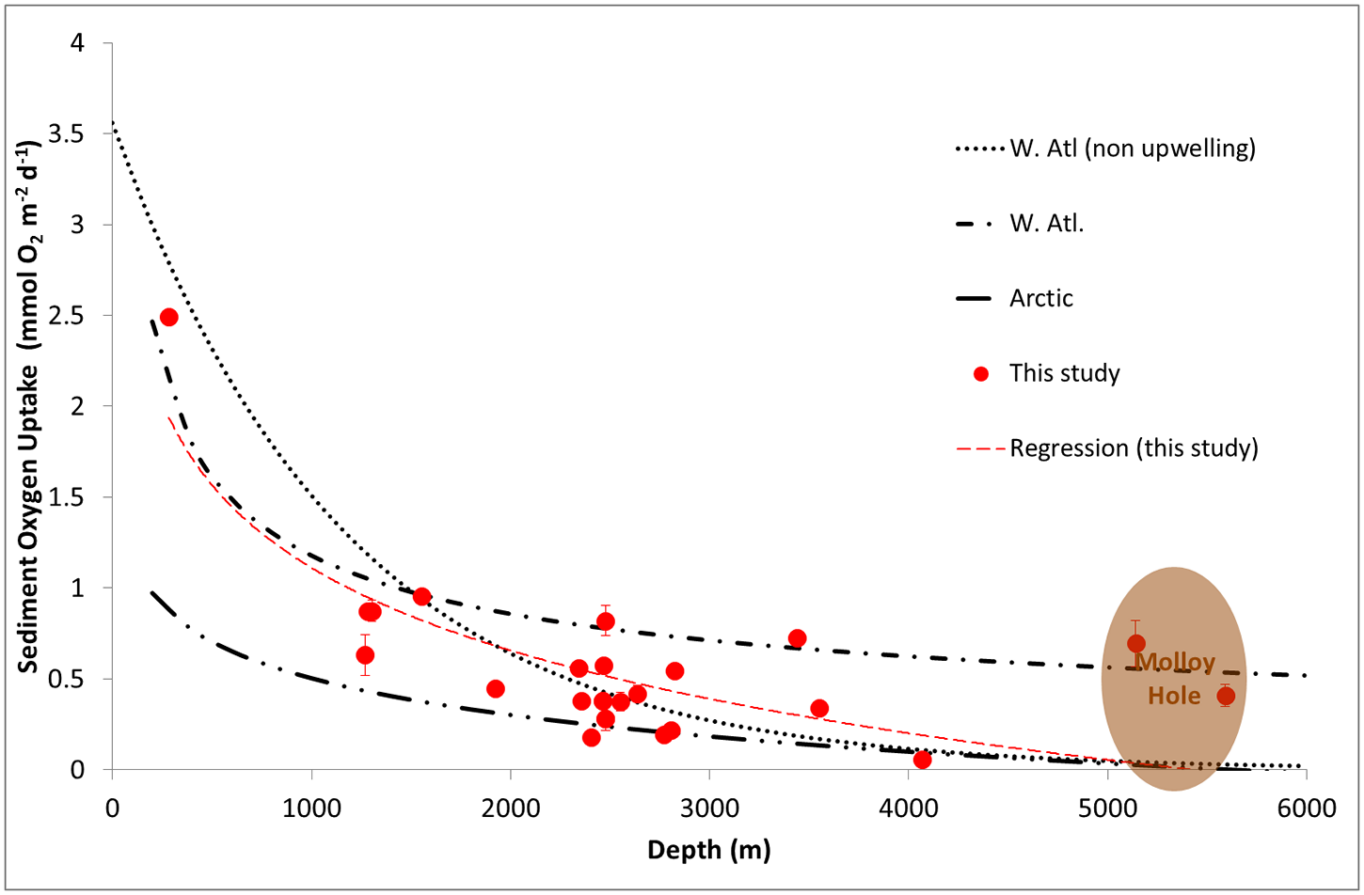
Sediment oxygen uptake plotted as a function of water depth. Stations from the HAUSGARTEN site and the associated logarithmic regression are plotted along with a compilation of several datasets and relationships available in the literature for the Arctic and Atlantic Ocean. Dotted line indicate data from [53]. Single dot—dash line indicate data from [22]. Finally data from [50] are plotted as a dashed line. Our regression appear as a red dashed line (y = -0.659 ln(x) + 5.6683, r² = 0.6441)

Down the continental slope, the mineralization of degradable organic matter declines from the shelf edge towards the central Fram Strait and shows an increase in the Molloy Hole. The overall decrease with increasing water depth is a common feature in margin sediments [22, 53]; the increase at great depth is a feature which has been observed in other deep trenches [54] and will be discussed at the end of the paragraph. Ignoring the deepest stations along the bathymetric transect, all our surface sediment descriptors (pigment, phospholipid, and protein contents, organic carbon contents, see Table 2) show an exponential decrease along the depth transect. Such a decrease is directly reflected in the O_2_ uptake (Fig 2), which follows a significant exponential decrease with water depth (r^2^ = 0.644, p < 0.05, Fig 6). This negative correlation with water depth is consistent with previous empirical relationships originating from compilation of data sets in the Arctic and the Atlantic Ocean (Fig 6) [22, 50, 53, 55, 56]. TOU, measured with an *in situ* benthic chamber on 3 stations only, displays the same behavior (P. Hall unpublished data) with a decrease by a factor of 2 between 1000 and 2500 m depth and an increase in the Molloy Deep compared to the site at 2500 m. Due to the limited number of TOU and thus the absence of a clear overview of the TOU/DOU distribution along the entire transect we used DOU as a proxy for carbon remineralization in this area. Nevertheless, we acknowledge that the decrease in oxygen consumption rates versus depth might hence be underestimated by ignoring the contribution of the benthic fauna. Previously documented across continental shelves and slopes at various latitudes, such decrease in benthic degradation activity with increasing water depth is a known feature associated with a decrease in benthic faunal abundances [57] and related to the amount of organic matter reaching the seafloor. Although the organic carbon contents measured in this study are in the lower range for values generally reported at these depths [54, 58, 59], remineralization fluxes correspond well to values published for other non-upwelling margins [60–62].

**Fig 7:**
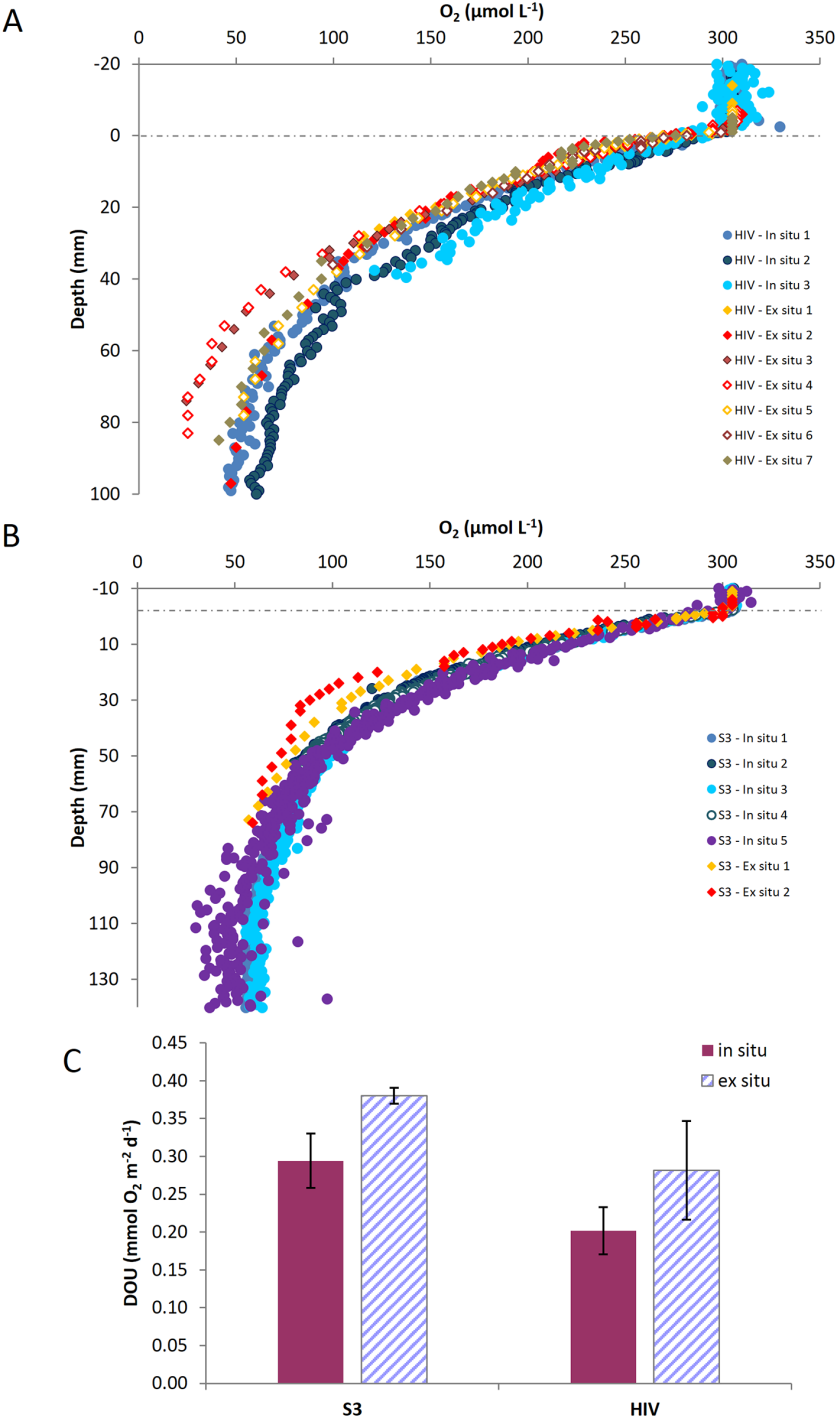
Comparison of *in situ* and *ex situ* oxygen microprofiles and sediment oxygen uptake. *In situ* and *ex situ* oxygen microprofiles at station HGIV (**A**) and S3 (**B**). Comparison of *in situ* vs *ex situ* diffusive sediment oxygen uptake (**C**).

Given the variations in sampling depth, one could question the effect of core recovery on the fluxes measured from on board profiling. In 2007 at stations S3 and HIV, we performed both in situ and ex situ measurements, allowing us to infer potential biases in our DOU estimates. Overall, laboratory measurements on recovered sediment cores showed steeper O_2_ gradients by 30% (we did not reach the oxygen penetration depth) and a higher diffusive uptake than *in situ* measurements (Fig 7). Such observation is quite common and likely result from a transiently increased temperature during recovery, DOC release through cell lysis and enhanced microbial activity in decompressed sediment cores [25, 63]. Differences between in situ and ex situ values increase with water depth. Hence, our onboard fluxes might be overestimated compared to absolute values. Nevertheless, such overestimation should not be a problem when comparing the DOU of sediments along a latitudinal transect (i.e. same water depth). Along the bathymetric transect, however, such bias might affect the observed trends. Nevertheless, as specified above, our measured DOU values match well those published for similar deep-sea environments. In addition, our gradient along the bathymetric transect follows previously documented bathymetric relationships, giving us confidence in our DOU data.

As observed by Boetius & Damm [50] in the Laptev Sea, labile organic carbon availability and associated benthic mineralization activity along the bathymetric transect in the HAUSGARTEN area are likely driven by incoming POM. Indeed, along the slope, our measured DOU values are very close to the POM fluxes at depth predicted from the empirical relationships from Berger et al. [64] and the modeled annual primary production rate of Skogen et al. [47] in the Greenland Sea. As evidenced by very low pigment (Chl *a* and Pheo), protein, and organic carbon (C Org) contents at the deeper stations (3500–5590 m water depth; Table 2), the low amount of labile primary produced carbon reaching the seafloor seems to be efficiently degraded and processed within the benthic ecosystem, emphasizing the low burial within deep-sea environments [65, 66].

It is noteworthy to mention that stations along the bathymetric transect might display different sedimentation rates with direct impact on the age of the sediment integrated over 5 cm, and its potential bioavailability. Higher sedimentation rates at the shallower stations would lead to fresher material in the top 5 cm, and potentially higher degradation activities. The decrease of our biogeochemical descriptors and the measured benthic oxygen uptake with depth may hence also reflect the deposition timescale of the sediment [67].

The shelf edge, the upper slope and the Molloy Hole stations show higher DOU compared to other stations along the bathymetric transect (Fig 6). The high rates measured at the shallow sites reflect both the high coastal primary production and a possible lateral input of organic matter from the Arctic fjords [56]. The high sediment oxygen uptake observed in the Molloy Hole, compared to typical DOU at similar water depths (Fig 6), indicates lateral advection of organic matter at this site, also through large eddies and turbidites [68, 69]: the Molloy Hole seems to act as a deposit center for organic matter. At a regional scale, this large dip into the Greenland Sea concentrates the organic matter both produced in surface waters and advected down the continental slope, making this >5000 m deep site a unique feature in the Arctic Ocean as active as a 2500 m deep site in terms of DOU.

### Benthic mineralization along the latitudinal HAUSGARTEN transect

The productivity and pelagic-benthic coupling of the Arctic Ocean largely rely on the extent, thickness and melting dynamics of sea ice, but also on the river discharges and inflow of Atlantic waters [7]. Our study site, the HAUSGARTEN area, is located in the Fram Strait, a transition zone between the North Atlantic and the central Arctic Ocean: our dataset thus offers a unique opportunity to investigate the spatial variations of the benthic mineralization at a local scale in an ice-edge region.

To investigate the effect of the ice-edge on the benthic degradation of organic carbon, we focused on a latitudinal transect along the 2500 m isobath, with stations at increasing distances to the sea ice. Overall, the DOU measured along this approx. 150 km long transect were similar to previously published values for Arctic sediments at similar water depth [50, 70, 71]. Over the two years 2007 and 2008, DOU show lower values close to the ice-edge and an increase towards the southern stations (Fig 3). In addition, PCA results indicate strong differences in biogeochemical sediment descriptors along the latitudinal transect (Fig 4). Generally, the latitudinal transect is associated with lower organic contents and bacterial activity near the ice-edge in the North and differences in the organic matter quality between northern and southern stations (protein vs. phospholipid contents). The southern sites, located in the ice-free region, are distinct from the northern ones, with the northern most station (N4) being clearly disparate, which suggests different benthic processes and food inputs along this latitudinal transect. Singularly, the northern most station N4 seems to be characterized by low amounts of organic matter of good quality as indicated by slightly increased protein contents and higher proportions of chlorophyll contents (%Chl *a*).

DOU and esterase activity represent respectively the total benthic carbon degradation activity, and the metabolic potential of bacteria, and are hence proxies for the degradation in the sediments of respectively total and intermediate-reactivity organic matter [72, 73]. Both quantities showed larger values in the South compared to the Northern section of the transect, which indicates that the HAUSGARTEN benthic ecosystem along the latitudinal transect depends on the quantity rather than the quality of organic matter reaching the seafloor. Our benthic data hence suggests a larger deposition of organic matter of lower lability in the southern section of the transect and a lower quantity of organic matter with higher quality in the North, near the ice edge.

The satellite-derived surface chlorophyll maps (Fig 5) indicate consistent gradients for June, July, and August with larger Chl *a* concentrations in the Southern part of the HAUSGARTEN area (Fig 5, white box) compared to the North, suggesting that the inflowing Atlantic waters sustain primary production in the South over the summer. This is clearly not the case in the northern part of the transect where surface Chl *a* during the summer is low. Contrastingly, the month of May shows higher Chl *a* content in the ice-retreating zone (northernmost part of the section) compared to the South where the phytoplankton bloom has not yet started. Such production pattern is typical of an ice-retreating zone where stratification due to ice melting promotes primary production [74]. This type of seasonal production generally promotes large diatom blooms which are exported rapidly at depth [75, 76] exhausting nutrients for the rest of the seasonal production, which is consequently small. There seems thus to be two production regimes: the Atlantic water regime with continuous primary production over the summer accompanied by low and continuous grazing [42] and the ice-edge regime with a sudden increase of primary production in the spring (May) followed by large and temporary export of labile organic matter and little production during the summer. It has been shown [27] that the duration of the production season is the main parameter for total production and export and it is thus expected that over the year, the southern part of the Fram Strait exports more organic material than the Northern section.

Although it is established that the link between primary production in the Fram Strait and the biogenic matter rain rate is quantitatively limited with an export of around 2% of the production [77], our benthic results match such a production regime with a larger quantity of more refractory organic matter in the South (export by grazing which lowers the organic matter quality as it is transferred in the food chain, recycled and degraded in the water column[78]) and a smaller quantity of more labile organic matter in the North (export by diatom aggregations?[79]). It is noteworthy that the DOU at the central HAUSGARTEN station (HG IV; 0.33 ± 0.07 mmol O_2_ m^-2^ d^-1^) matches very well the downward POC flux measured during summer by sediment traps over 3 years by Bauerfeind et al. (2009; 0.42 ± 0.07 mmol C m^-2^ d^-1^). This indicates that the degradation of settling material in the water column between 300 and 2500 m is limited and that the sediment deposition flux mimics the export from the upper water column.

The projected increase in primary production in the Arctic Ocean due to the incursions of warm Atlantic Waters during global warming would thus suggest that a transition towards larger export of more refractory organic matter is conceivable. This larger export would be mainly due to the longer production season linked to the extension of the ice-free zone.

By showing an increased POC flux to the sediment as reflected by DOU and a more refractory material as reflected by less labile organic matter, our benthic mineralization rates highlight important features of the carbon cycle and benthic—pelagic coupling in the Arctic Ocean: Atlantic water inflows have a direct influence on local seasonal production, ice extent and vertical flux. Such observations are valuable and can be used to evaluate the predicted effects of climate change in the Arctic Ocean, which include the shift from a polar to an Atlantic dominated regime, and from a multiyear ice, to a more seasonal ice zone and open water-dominated environment [80], and to further assess the effects of such changes on arctic benthic ecosystems.
